# The Effect of Ethanol on Gelation, Nanoscopic, and Macroscopic Properties of Serum Albumin Hydrogels

**DOI:** 10.3390/molecules25081927

**Published:** 2020-04-21

**Authors:** Seyed Hamidreza Arabi, David Haselberger, Dariush Hinderberger

**Affiliations:** Institut für Chemie, Martin-Luther-Universität Halle-Wittenberg, Von-Danckelmann-Platz 4, 06120 Halle, Germany; seyed.arabi@chemie.uni-halle.de (S.H.A.); david.haselberger@student.uni-halle.de (D.H.)

**Keywords:** serum albumin, hydrogels, drug delivery, materials science, protein denaturation

## Abstract

Serum albumin has shown great potential in the development of new biomaterials for drug delivery systems. Different methods have been proposed to synthesis hydrogels out of serum albumin. It has been observed that ethanol can also act as a trigger for serum albumin denaturation and subsequent gelation. In this study, we focus on basic mechanisms of the albumin gelation process at 37 °C when using the chemical denaturant ethanol. The temperature of 37 °C was chosen to resemble human body temperature, and as under physiological conditions, albumin is in a non-denatured N conformation. As established in our previous publication for the triggers of pH and temperature (and time), we here explore the conformational and physical properties space of albumin hydrogels when they are ethanol-induced and show that the use of ethanol can be advisable for certain gel properties on the nanoscopic and macroscopic scale. To this end, we combine spectroscopic and mechanically (rheology) based data for characterizing the gels. We also study the gels′ binding capacities for fatty acids with electron paramagnetic resonance (EPR) spectroscopy, which implies observing the effects of bound stearic acids on gelation. Ethanol reduces the fraction of the strongly bound FAs in bovine serum albumin (BSA) hydrogels up to 52% and induces BSA hydrogels with a maximum storage modulus of 5000 Pa. The loosely bound FAs in ethanol-based hydrogels, besides their relatively weak mechanical properties, introduce interesting new materials for fast drug delivery systems and beyond.

## 1. Introduction

Serum albumin is the most abundant protein in vertebrate blood plasma with typical concentrations of 35–45 mg/mL (0.53–0.68 mM) [1]. It is essential for the oncotic pressure and serves as carrier for many hydrophobic substances in the blood plasma [1]. It has the molar mass of approximately 66.000 Da [2]) and consists on average of 585 amino acids [3]. Due to its availability at high purity and relatively low price, serum albumin is used for many physicochemical and biochemical studies [3,4]. Human serum albumin (HSA) is mainly used for medical, metabolism and genetic research [3]. Bovine serum albumin (BSA) shares 75.52% of its primary structure with HSA [3]. It is used as a model protein for in vitro tasks [3]. In a previous study, we have shown that HSA and BSA both display a complex and rich phase behavior that includes very interesting methods pioneered by Baler et al. of achieving protein hydrogels based on thermal denaturation or triggering electrostatic interaction networks through changes in pH [5,6].

Serum albumin has shown promising properties to develop new biomaterials for drug delivery systems [5]. Through thermal or chemical methods and at the cost of loss of native α-helices in the structure, there is a potential to form intermolecular β-sheets leading to a cage-like structure in which water or other molecules can be trapped [6]. The goal is to synthesize gels with high stability and biocompatibility, and the luxury of being able to tailor the properties based on certain requirements.

In our previous work [6], the properties of bovine, and for the first time human, serum albumin (BSA and HSA, respectively) gels generated through tuning of pH or temperature as established by Baler et al. [5] have been extensively studied. The incubation time of the gels has been introduced as an excellent parameter for fine-tuning of gel properties and the data were summarized in phase diagrams [6]. Starting from this point, in preliminary tests, gel formation has been observed in a mixture with a wide variety of different BSA and ethanol concentrations after an incubation time of 16 h at 37 °C. To understand the properties of these hydrogels and their differences compared to hydrogels made based on other methods (pH- and temperature-induced), different aspects have been systematically investigated, namely (i) at which concentrations of bovine serum albumin and ethanol hydrogels form, (ii) how these different concentrations affect the mechanical stability of the gels, (iii) what molecular structure changes are observable depending on time, (iv) how stearic acids affect the gelation process, and (v) how the fatty acid (FA) binding capacity of the hydrogels changes. Fatty acids are chosen as there are seven FA binding sites in albumin that are known to stabilize the tertiary structure of individual albumin proteins [7,8].

To achieve this, the rheological properties during the gelation process are measured, the changes in secondary structure are characterized using their IR-spectroscopic signatures, and a nanoscopic view on the fatty acid (FA) binding capacity of albumin hydrogels by means of electron paramagnetic resonance (EPR) spectroscopy, both of which have been established before [6].

## 2. Results

The gelation experiments have been performed in several experimental setups, generally in a thermomixer (under shaking, see Materials and Methods). Gel formation has then also been studied in the rheometer for rheological experiments, in the IR spectrometer (ATR setup), and the EPR spectrometer during the respective spectroscopic measurements.

### 2.1. General Gelation Properties

**Figure 1:**
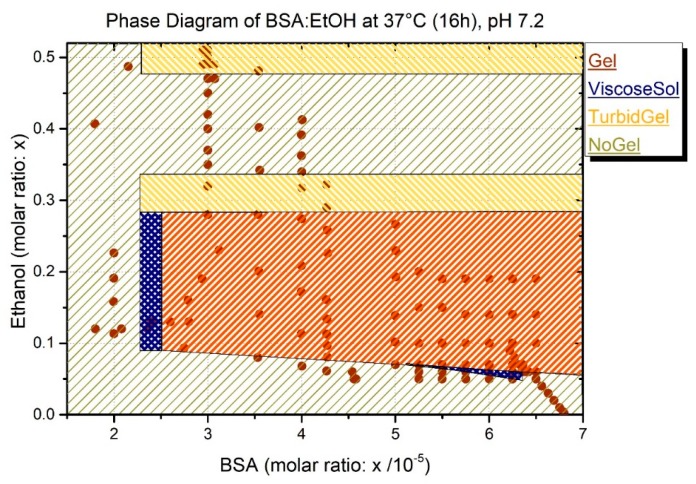
Phase diagram of bovine serum albumin (BSA) after 16 h at 37 °C (data points based on Table A1). ViscosSol refers to viscous solution but fall short of being a robust gel. Gel refers to clear gel which can be differentiated from turbid gels. NoGel refers to state like precursor solution (not viscous). Table A1 (see Appendix A), which shows the details of the exact albumin and ethanol concentration for each point shown in Figure 1. From these data, it is apparent that the formation of hydrogels using ethanol is correlated to both the concentration of ethanol and the concentration of BSA. The concentration of BSA can be in some cases lower (compared to samples where no gel formation is observed) where due to the presence of a higher amount of ethanol hydrogels form; yet, the BSA concentration should be above the threshold of 7.15 wt% (1.07 mM) for a three-dimensional gel to form. The results of the gelation in the thermomixer show that the minimum amount of ethanol cannot be trivially found and is not dependent on a certain amino acid to ethanol (AA/EtOH)-ratio. Gels were e.g., formed with an amino acid to ethanol ratio of 1.9, but other samples did not form gels at a very high (AA/EtOH) ratio of 11.6.

As shown in Figure 1, a wide variety of the different BSA and ethanol concentrations displays a complex phase diagram after an incubation time of 16 h at a temperature of 37 °C. All gelation experiments were performed at a neutral pH (approximately 7.2), allowing an overall mild environment. This comes at the cost of mechanical stability and binding capacity compared to the other methods, putting more chemical or thermal stress on the protein solutions [6].

Figure 1 illustrates the property (ethanol mole fraction versus BSA mole fraction) space of samples prepared at 37 °C after an incubation time of 16 h. The mole fractions were used to remove the effect of volume dilution that would have to be taken into account for the concentrations. As mentioned above, there also seems to be a minimum amount of BSA needed for the gelation. Below this concentration (1.07 mM), the proteins cannot form enough cross-links to build a gel, potentially because of low probabilities for intermolecular secondary structure (β-sheet) formation. The absolute values (without ethanol) for gel formation have been extensively studied in a previous publication [6] to find the absolute minimum with ethanol; more experiments are needed and are beyond the scope of this study. Another interesting phenomenon can be observed by the addition of high amounts of ethanol. At molar fractions between 0.3 and 0.35 of ethanol, the obtained gels show a high turbidity. After adding more ethanol, only a turbid liquid is obtained. Nevertheless, above a molar fraction of 0.49, turbid gels can be observed again. The turbidity reflects the inhomogeneity of the gel structure.

### 2.2. Rheological Characterization

The combined results obtained from rheological measurements of the gelation process on the rheometer plate show that ethanol plays a significant role in increasing the gelation rate, even at low protein concentrations. As a reference, one can state that no gelation can be observed at 37 °C and neutral pH regardless of BSA concentration [5,6]. By setting the pH to more extreme values (lower than 4.2 or higher than 10.6), hydrogels form, as described by e.g., Baler et al. and us [5,6].

**Figure 2:**
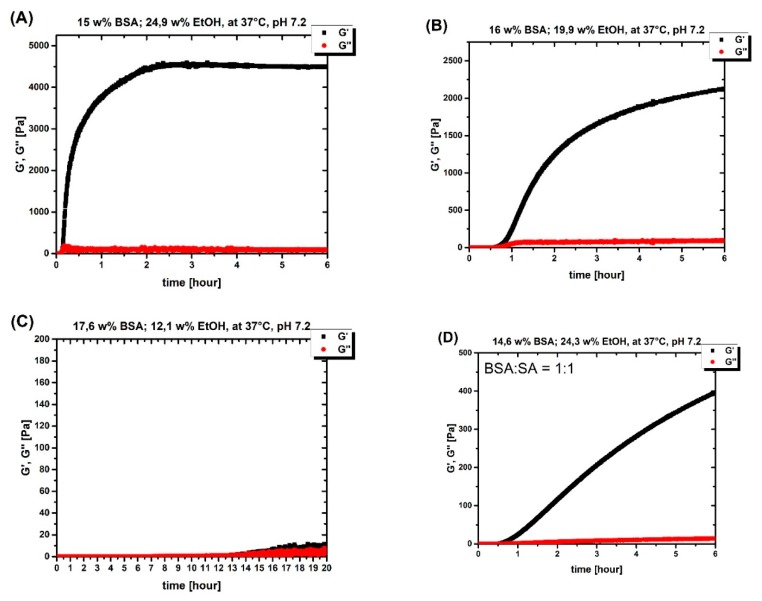
(**A**) 15 wt % (2.2 mM) BSA solution with 24.9 wt % EtOH at 37 °C on a rheometer plate for 6 h. (**B**) 16 wt % (2.4 mM) BSA solution with 19.9 wt % EtOH at 37 °C on a rheometer plate for 6 h. (**C**) 17.6 wt % (2.6 mM) BSA solution with 12.1 wt % EtOH at 37 °C on a rheometer plate for 20 h. (**D**) 14.6 wt % (2.1 mM) BSA solution with 24.3 wt % EtOH at 37 °C on a rheometer plate for 6 h with the BSA: stearic acid (SA) ratio of 1:1.

**Figure A1:**
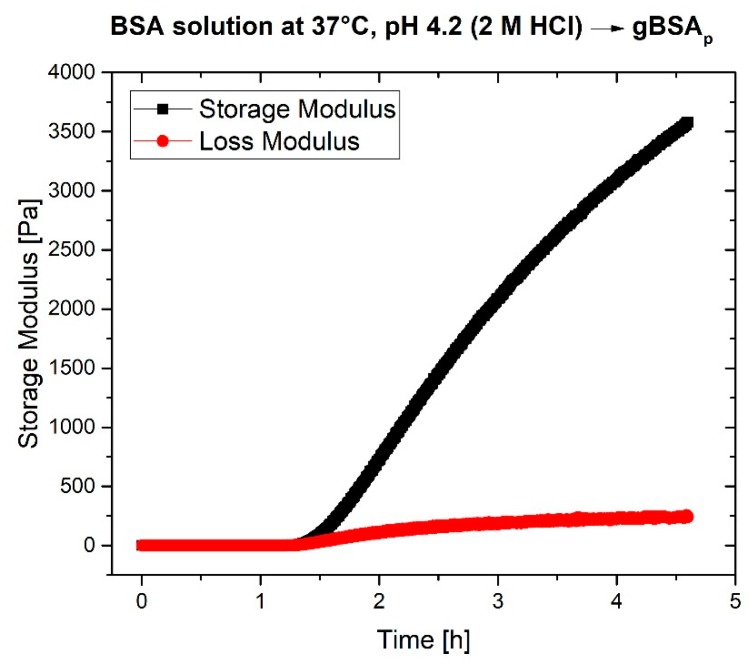
3 mM BSA solution with no EtOH at 37 °C on rheometer plate. The pH is set to 4.2 by using 2M HCl.

In the experiments presented here using ethanol, the gelation proceeds much faster at 37 °C and neutral pH than when the pH is lowered to 4.2 or lower (see (Figure 2) and (Figure A1)). The rate of ethanol-induced gelation is comparable with that of high pH value gelation processes. To translate the measured values of G′ and G’’ into a comprehensible concise graph, the measured points in the diagram of Figure 1 are classified as (i) gel, (ii) no gel, (iii) turbid gel, and (iv) viscous solution (the classification is based on the gel classification in ref. [6]).

To elucidate the mechanical properties and the rate of gelation of the gels obtained from ethanol addition, the results are compared with the other hydrogels synthesized with other methods. Table 1 shows that the addition of ethanol, despite lower protein concentration, paves the way of gel formation to a more robust and more rapid gelation compared to the pH-induced method.

Furthermore, Figure 2 (see also Figure A3) illustrates the correlation between BSA and ethanol concentration; in Figure 2A, although the protein concentration is lower compared to Figure 2B, due to the higher ethanol concentration, gelation starts earlier and after six hours, the G′ (and the G’, G’’ difference) is higher. Figure 2C shows the importance of the role that EtOH plays, a higher BSA concentration results in no gelation after 20 h when the ethanol concentration was not high enough.

**Figure A2:**
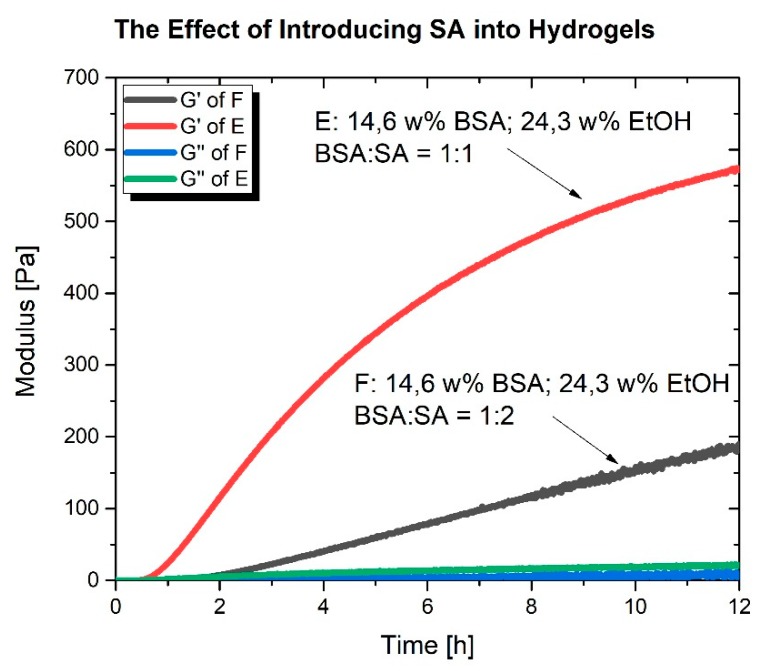
Addition of stearic acid (SA) into the precursor solution of the gels (2.1 mM BSA solution) leads to weakening the mechanical properties of the gels. The SA were dissolved in 0.1 M KOH prior to addition into precursor solution.

**Figure A3:**
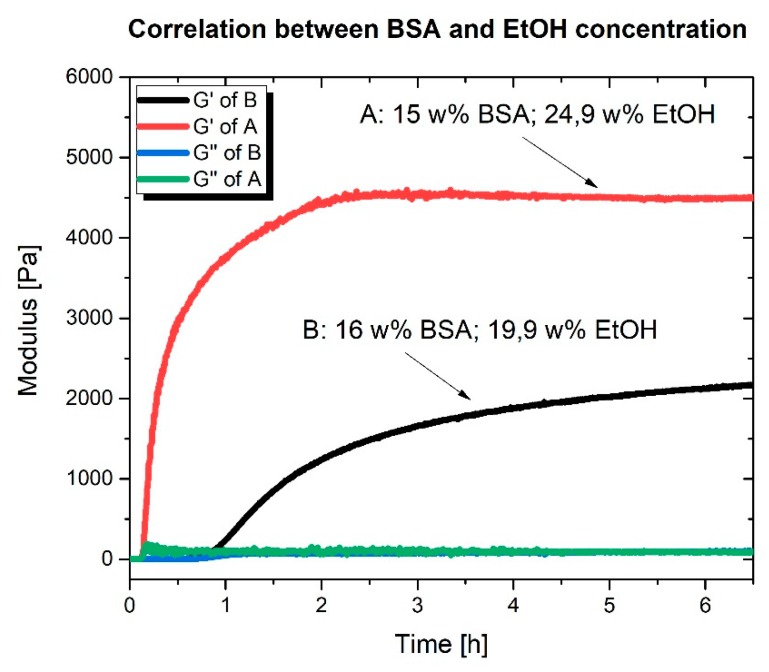
This figure illustrates that there is a correlation between ethanol and protein concentration. In A, although the BSA concentration is lower, due to the presence of more ethanol, a mechanically more robust gel is formed. In B, still a gel forms, but a higher concentration of BSA does not lead to a stronger gel structure, since the amount of ethanol is lower compared to A.

To investigate how the addition of stearic acid (SA) affects the gelation properties, gels were made on the rheometer plate in the presence of SA at different BSA:SA molar ratios. Figure 2d shows the adverse effect of SA on the mechanical properties of the gel. The more SA present, the less robust the gel (see Figure A2). This observation is also made without ethanol (see [6]) and can be traced back to the stabilizing effect of bound SA on the tertiary structure of individual albumin molecules. In addition, the denaturation temperature increases when SA ligands are bound [9].

As outlined above, to obtain more molecular/nanoscopic insights into the macroscopic/rheological properties of the gel and the gelation processes, the gelation process at the molecular level using IR and continuous wave (CW) EPR spectroscopies was explored.

### 2.3. Infrared Spectroscopy (IR)

**Figure 3:**
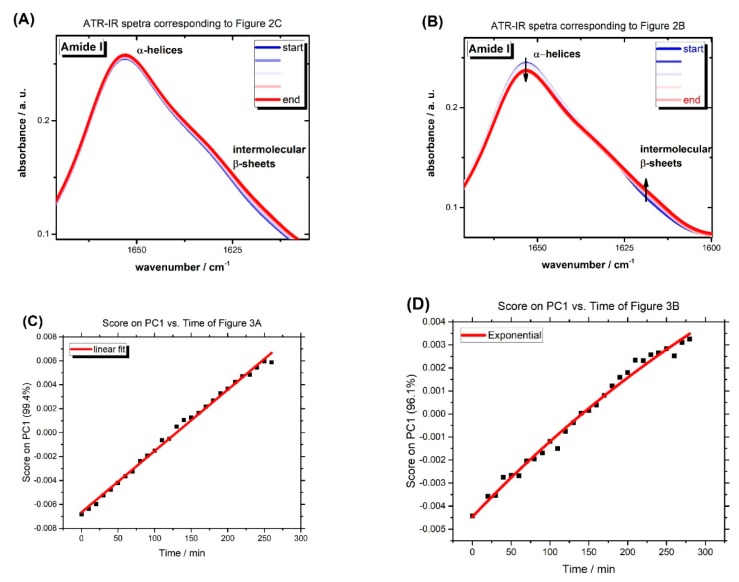
(**A**) Amide 1 band of 2.6 mM BSA solution with 12.1 wt % EtOH at 37 °C on ATR-IR crystal (BioATRCell), the same condition as Figure 2C, shows homogenous changes across all the amide 1 band and no gel formation. (**B**) Amide 1 band of 2.4 mM BSA solution with 19.9 wt % EtOH at 37 °C on ATR-IR crystal, the same condition as that in Figure 2B, shows an increase in the intermolecular β-sheet peak, whereas native α-helices of BSA are lost. In this case, gel formed on the spectrometer crystal. (**C**) PC1 scores vs. time regarding Figure 3A, (**D**) PC1 scores vs. time regarding Figure 3B. For more detailed information about PCA analysis, see ref. [6].

It has been shown [6] that the formation of intermolecular β-sheets at the cost of losing native α-helices leads to gel formation. This process is detectable by recording the spectra during gel formation on the crystal of an attenuatated total reflection infrared (ATR-IR) spectrometer (A Bruker Tensor 27 FT-IR spectrometer equipped with a BioATRCell II and an LN-MCT photovoltaic detector and the OPUS Data Collection Program (all from Bruker, Ettlingen, Germany)) [6]. When ethanol and BSA concentrations are in the range where no gelation can be observed macroscopically (see Figure 1), there are also no significant time-dependent changes detectable in the IR spectra (Figure 3A). In contrast, in experiments showing gel formation macroscopically and in rheology, there is an obvious increase in intermolecular β-sheets at the cost of a decrease in native α-helices (Figure 3B).

To find the correlation of the recorded IR spectra during the gelation, PCA (Principal Component Analysis) was applied to the spectral range of 1600–1700 cm^−1^ after the subtraction of a linear baseline and vector normalization of the spectra. In contrast to the linear changes in PC1 (first principal component), where more than the 95% of possible variance is detected, of Figure 3A versus time (see Figure 3C), Figure 3D shows exponential changes during the gel formation of Figure 3B. These results show that as long as no hydrogel formation takes place, changes on the nanoscopic level (secondary structure) are detectable, linear, and in all regions with identical effect, while concerted, yet strongly inhomogeneous changes during gel formation can be detected.

### 2.4. Electron Paramagnetic Resonance (EPR) Spectroscopy

**Figure 4:**
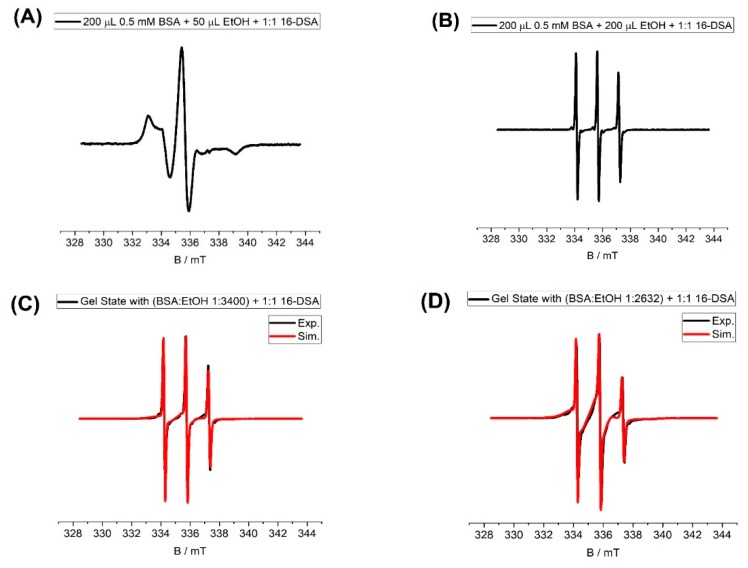
The samples are prepared based on Table A2. Electron paramagnetic resonance (EPR) spectra of (**A**,**B**) shows that the addition of ethanol dramatically weakens the interaction between protein and stearic acid. The data for (**C**,**D**) and Figure A4 are shown in Table 2.

The performed EPR spectroscopic measurements show the significance of ethanol concentration on the protein structure and the FA binding sites that are predominantly situated at the interfaces of α-helices (Figure 4a). For EPR spectroscopy, as generally established, stearic acids were employed that have an attached persistent nitroxide radical group, most often at position 16 of the alkyl chain (16-doxylstearic acid, 16-DSA) [5,7,10]. Generally, the EPR data indicate that when keeping the BSA and 16-DSA concentrations constant and merely increasing the amount of ethanol, fatty acids tumble much faster (see Table A2). This can be interpreted when one considers that for the amphiphilic 16-DSA molecules, ethanol in fact is a good solvent, and as such competes with binding to the—in water or buffer—much more favorable FA binding sites. Overall, the interactions between BSA and FAs are strongly weakened with significant amounts of ethanol present in the solutions/gels.

The comparison between (Figure 4B) with the EPR results obtained from other BSA hydrogels, synthesized by other methods, shows that the addition of ethanol results in weaker interaction between the BSA and FAs and consequently the faster tumbling of FAs. Accordingly, the results of EPR measurements reveal that the ethanol-induced gelation leads to a lower fatty acid binding capacity compared to other gelation methods [6]. They indicate that the amount of bound fatty acids relative to the amount of overall available fatty acids is decreasing with higher ethanol concentrations, but it does not change with higher fatty acid concentration in the tested concentration range, implying a thermodynamic equilibrium of bound and free fatty acids in the gelation process.

## 3. Discussion

Our experiments show that the addition of ethanol results in gel formation at low temperature and neutral pH values. The correlation between ethanol and albumin concentration for gel formation is remarkable and detectable at macroscopic and molecular/nanoscopic levels. These results are concisely summarized in the phase diagram of Figure 1. When the ratio between EtOH and BSA is not in the range of gel formation, there is no sign of formation of intermolecular β-sheet (See Figure 3A). Although in ethanol-induced gels, the minimum concentration of albumin for gel formation is lower compared to the previously established pH-induced method, when the protein concentration is too low (below the gel formation threshold), the addition of EtOH cannot compensate for the low concentration of protein. On the other hand, when there is too much EtOH (see Figure 1) in the solution, it effectively denatures and solvates the proteins and as such has an adverse effect on the formation of the intermolecular β-sheet. Therefore, there is range of BSA:EtOH ratios, in which the formation of 3D-linked hydrogels of albumin molecules is possible. This cage-like network of protein is capable of holding water inside. As with temperature-induced gels, this comes at the cost of losing extensive amount of native α-helices in BSA (see Figure 3B). That means that the secondary structure of ethanol-induced hydrogels are comparable with temperature-induced ones.

Based on the rheological results, the mechanical properties of the gels are significantly poorer compared to pH-induced and temperature-induced hydrogels (see Table 1 and Figure 2). On the other hand, gel formation proceeds significantly faster in comparison to pH-induced hydrogels. These results are substantiated with EPR measurements; namely, the number of binding sites are remarkably reduced compared to other gelation methods, including temperature-induced methods [6]. Despite the strong denaturation of albumin during temperature-induced gelation, the resulting hydrogel is mechanically very robust (see ref. [6]), which can be concluded that although fatty acids will not be bound to hydrogel scaffold (protein wall), the FAs are rather trapped in strongly demobilized water phase of the hydrogels. Moreover, in the pH-induced method, despite lower rigidity (the mechanical properties are weak in comparison to temperature-induced gels), more of the native secondary structure of albumin is preserved, and that results in a relatively high number of available FA binding sites (see ref. [6]).

Hence, comparing the EPR and rheological results for the ethanol addition method and other methods shows that with ethanol addition, the protein has weaker interactions (less binding capacity) with Fas, and the water phase is less immobilized compared to the temperature-induced method. This can be of interest for fast drug release systems.

We hope that this initial study, which connects the nanoscopic properties with the macroscopic features of the hydrogels leading to phase diagrams, will pave the way also for their exploration in applications such as the controlled release of (bio-)molecules.

## 4. Materials and Methods

### 4.1. Materials and Gel Preparation

Human (HSA) and bovine (BSA) serum albumin were purchased from the spin-labeled fatty acid 16-DOXYL stearic acid, 16-DSA (16-doxyl stearic acid, free radical, 253596, ALDRICH, Sigma-Aldrich Chemie GmbH, Schnelldorf, Germany). All chemicals and materials were used as received without further purification. Hydrogels were prepared in a thermomixer made by Halle University workshop (Halle, Germany).

### 4.2. Gel Formation

The precursor solutions were prepared by dissolving serum albumin (BSA) in water. The amounts were chosen based on the desired concentration. After two hours of stirring and the complete dissolution of BSA in water, ethanol (EtOH) is added. The samples and the exact amount of each component are tabulated in Table A1.

### 4.3. Loading Stearic Acid (SA) into Gels

Stearic acid is dissolved first in 0.1 M KOH, and then it is added into the precursor solution of the gels. In these cases, the EtOH was added after the addition of the SA.

### 4.4. Rheology

Hydrogels have viscoelastic behavior under force load, i.e., the lattice stores the energy, which is retractable (elasticity), while some energy is dissipated. To describe this behavior, the storage- and loss-modulus are measured with an Anton Paar Physica MCR 301 rheometer (Anton Paar, Graz, Austria). The storage modulus represents the elastic portion (stored energy), and the loss modulus represents the viscous characteristics (energy dissipation). They are defined through Equations (1) and (2). For ideal elastic behavior, G′ >> G’’, and for ideal viscous behavior, G′ << G’’.

(1) $$Storage\;Modulus\;G^{\prime }:\;\;\;\;\;\;\;\;\;\;\;\;\;\;\;\;\;\;\;G^{\prime }=\frac{\tau_{A}}{\gamma_{A}}\;Cos\delta$$

(2) $$Loss\;Modulus\;G^{\prime \prime }:\;\;\;\;\;\;\;\;\;\;\;\;\;\;\;\;\;\;\;\;\;G^{’’}=\frac{\tau_{A}}{\gamma_{A}}\;Sin\delta$$

τ/Pa = shear stress

γ/% = deformation

δ/° = phase shift angle

In the gelation process, the values of G′ and G’’ are important. In the sol state, G’’ > G’, at the gelation point, G’’ = G’, and in the gel state, G′ > G’’. Based on the extent of the difference between G′ and G’’, we define mechanically strong and weak hydrogels. These definitions all take the magnitude of G’’ into account, which means that despite there being a large difference between G′ and G’’, the mechanical properties may still be poor when G’’ still is very high.

### 4.5. Infrared Spectroscopy (IR)

The secondary structure of proteins can be characterized using vibrational (infrared and Raman) spectroscopy. Infrared spectroscopy is based on the interaction of infrared radiation with molecules [10]. The IR radiation of certain wavelengths excite the vibrational modes of specific bonds whose wavelength and peak shape often depend on the local secondary structure. As a result, the IR absorption spectrum gives information on the secondary structure of the sample. The absorbance is plotted against the wavenumber (cm^−1^). In the scope of our study, the amide I band (between 1600 and 1700 cm^−1^) is the range of interest, since it shows the changes in the different secondary structures (α-helix, β-sheet, β-turn, and random coil) during the gelation [11]. The spectra were analyzed using Principal Component Analysis (PCA). PCA is a technique that transforms a number of possibly intercorrelated variables into a smaller number of orthogonal variables called principal components. This is to find trends and patterns in large datasets [10]. The goal is to de-correlate the original data by finding the directions along which the variance is maximized and then use these directions to define the new basis [10].

### 4.6. Electron Paramagnetic Resonance (EPR) Spectroscopy

Continuous wave (CW) EPR spectroscopy was used to investigate the fatty acid binding capacity of the hydrogels. By monitoring the changes in the dynamics of nitroxyl spin-labeled fatty acids (16-DOXYL stearic acid, 16-DSA, nitroxyl moiety at position 16 in the alkyl chain) during the presence and absence of the protein and comparing these changes in the gel state (in which the protein is denatured to some extent) and native state (native BSA has 7 ± 1 fatty acid binding sites [8]), we investigated the number of available FA binding sites of the gels. By simulating the spectra (using the EasySpin program package for MATLAB, in which the Schneider–Freed model of slow and intermediate rotational motion is implemented [12]), we can obtain information about the electronic and molecular structures, self-assembly, and dynamics of paramagnetic samples [7].

The CW EPR spectra were recorded using a MiniScope MS400 (Magnettech, Berlin, Germany) spectrometer working at the X-band (a microwave frequency of approximately ν = 9.4 GHz and a magnetic field sweep of 15 mT centered at 340 mT).

Temperatures were adjusted with a Temperature Controller H03 (Magnettech) with an accuracy of approximately 0.2 °C. The exact frequency was recorded using a frequency counter (Racal Dana 2101, Neu-Isenburg, Germany). Absorption spectra were detected as a first-derivative spectra through field modulation with 100 kHz and an amplitude of x mT.

## Figures and Tables

**Table 1:** The difference in mechanical properties and gelation rate at 37 °C between pH-induced and EtOH-mediated gelation.

Type	BSA Concentration	G′ (Pa) *	G′′ (Pa) *	Gelation Point
EtOH (25 wt%)	15 wt% (2.2 mM)	5000	150	8 min
pH induced ^1^	20 wt% (3 mM)	900	130	45 min
Temp. induced	20 wt% (3 mM)	16000	300	1 min

^1^ pH-induced gelation: the pH value set to 3.5 using 2M HCl (See ref. [6]), * the value of G′ and G’’ after 2 h. In the EtOH-induced gelation, the G′ remains constant after 2 h; however, in pH-induced gelation, it increases up to 10,000 Pa. Temp. induced refers to temperature-induced gelation.

**Table 2:** Results of EPR samples of Figure 4 and Figure A4.

Sample	Fraction of Bound Fatty Acid	Correlation Time τ_c_ (ns)	Fraction of Free Fatty Acid	Correlation Time τ_c_ (ns)
C	68%	5.28	32%	0.22
D	78%	5.28	22%	0.22
E	68%	5.28	32%	0.22
F	48%	5.28	52%	0.22

* Samples preparation of (A) and (B) are not simulated.

**Figure A4:**
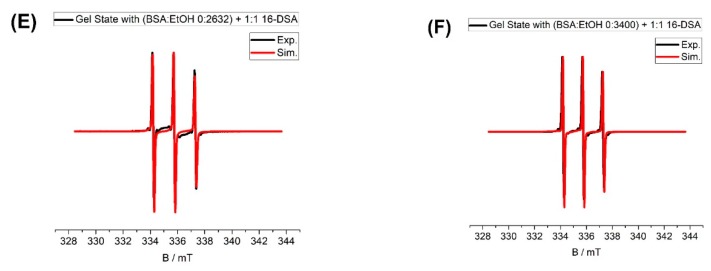
The samples are prepared based on Table A2. The data for (**E**,**F**) are shown in Table 2.

## Appendix A

**Table A1:** Samples and results after 16 h at 37 °C.

20 wt% BSA (μL)	EtOH (μL)	H_2_O (μL)	w% BSA	w% EtOH	x BSA 10^−5^	x EtOH	EtOH AA	Result
200	1	0	19.92	0.39	6.8	0.002	0.05	-
200	2	0	19.84	0.78	6.79	0.004	0.1	-
200	5	0	19.61	1.93	6.75	0.01	0.24	-
200	10	0	19.24	3.8	6.69	0.02	0.48	-
200	15	0	18.88	5.59	6.63	0.03	0.73	-
200	20	0	18.54	7.31	6.56	0.04	0.97	-
200	25	0	18.2	8.98	6.5	0.05	1.21	-
200	32	0	17.76	11.21	6.42	0.06	1.55	-
200	33	0	17.7	11.52	6.41	0.06	1.6	-
200	34	0	17.63	11.83	6.4	0.06	1.65	-
200	35	0	17.57	12.13	6.39	0.06	1.7	-
200	36	0	17.51	12.44	6.38	0.06	1.74	-
200	37	0	17.45	12.74	6.36	0.07	1.79	h. v.
200	38	0	17.39	13.04	6.35	0.07	1.84	gel
200	39	0	17.33	13.33	6.34	0.07	1.89	gel
200	40	0	17.27	13.63	6.33	0.07	1.94	gel
200	45	0	16.98	15.08	6.27	0.08	2.18	gel
200	50	0	16.7	16.48	6.22	0.09	2.42	gel
200	134.8	6.2	13.06	34.03	5.25	0.2	6.53	gel
200	96.5	8.6	14.49	26.74	5.5	0.15	4.68	gel
200	101.1	16.6	14.3	26.92	5.25	0.15	4.9	gel
200	61.5	10.7	16.1	18.72	5.75	0.1	2.98	gel
200	64.3	18.5	15.95	18.84	5.5	0.1	3.12	gel
200	67.4	27	15.8	18.98	5.25	0.1	3.27	gel
200	28.3	5.8	17.99	9.79	6.25	0.05	1.37	-
200	29.4	12.7	17.92	9.83	6	0.05	1.43	-
200	30.7	20.2	17.84	9.91	5.75	0.05	1.49	-
200	32.2	28.4	17.75	10.01	5.5	0.05	1.56	-
200	33.7	37.4	17.65	10.07	5.25	0.05	1.63	-
200	40.2	2.1	17.26	13.57	6.25	0.07	1.95	gel
200	41.9	8.89	17.16	13.66	6	0.07	2.03	gel
200	43.7	16.24	17.06	13.75	5.75	0.07	2.12	h. v.
200	45.7	24.3	16.95	13.85	5.5	0.07	2.22	h. v.
200	47.9	33	16.82	13.96	5.25	0.07	2.32	h. v.
200	38.5	2.66	17.36	13.04	6.25	0.07	1.87	gel
200	40.1	9.44	17.27	13.12	6	0.07	1.94	gel
200	41.9	16.8	17.16	13.23	5.75	0.07	2.03	gel
200	43.8	24.8	17.05	13.32	5.5	0.07	2.12	h. v.
200	45.8	33.7	16.94	13.39	5.25	0.07	2.22	-
200	36.8	3.19	17.46	12.5	6.25	0.06	1.78	gel
200	38.3	9.98	17.37	12.58	6	0.06	1.86	h. v.
200	40	17.4	17.27	12.68	5.75	0.06	1.94	-
200	41.8	25.4	17.17	12.76	5.5	0.06	2.03	-
200	43.8	34.3	17.05	12.85	5.25	0.06	2.12	-
200	40.6	17.2	17.24	12.85	5.75	0.07	1.97	gel
200	41.2	17	17.2	13.03	5.75	0.07	2	gel
200	50.3	42.7	16.69	14.05	5	0.07	2.44	gel
200	47.2	33.2	16.86	13.77	5.25	0.07	2.29	gel
200	46.5	33.4	16.9	13.58	5.25	0.07	2.25	-
200 *	28.5	0	3.48	10.11	4.58	0.05	1.95	-
200 *	29.3	0	13.45	10.36	4.57	0.05	2.01	-
200 *	30	0	13.41	10.58	4.56	0.05	2.05	-
200 *	33.5	0	13.25	11.67	4.54	0.06	2.29	-
200 **	136.1	17.8	15.38	33.02	6.5	0.19	5.04	gel
200 **	141.6	24.5	14.87	33.23	6.25	0.19	5.25	gel
200 **	147.5	31.8	14.36	33.42	6	0.19	5.48	gel
200 **	153.9	39.7	13.85	33.62	5.75	0.19	5.73	gel
200 **	160.9	48.3	13.32	33.83	5.5	0.19	6	gel
200 **	102	28.3	16.19	26.06	6.5	0.14	3.78	gel
200 **	106.2	35.4	15.66	26.25	6.25	0.14	3.94	gel
200 **	110.6	43.1	15.13	26.41	6	0.14	4.11	gel
200 **	115.4	51.6	14.59	26.57	5.75	0.14	4.29	gel
200 **	68	38.8	17.1	18.35	6.5	0.1	2.52	gel
200 **	70.8	46.4	16.54	18.48	6.25	0.1	2.63	gel
200 **	73.7	54.5	15.99	18.6	6	0.1	2.74	gel
200 **	34	49.3	18.11	9.72	6.5	0.05	1.26	-
200 **	46.3	45.5	17.73	12.95	6.5	0.06	1.71	gel
200 **	44.2	46.1	17.8	12.41	6.5	0.06	1.64	gel
90	84	110	6.76	24.89	2.36	0.12	9.05	h. v.
80	84	120	6.01	24.89	2.08	0.12	10.18	-
70	84	130	5.26	24.89	1.8	0.12	11.63	-
100	100	85	7.58	29.9	2.79	0.16	9.69	gel
100	115	70	7.67	34.8	2.94	0.19	11.15	gel
100	130	55	7.76	39.82	3.12	0.23	12.6	gel
100	162.2	51.8	7.15	45.74	3	0.28	15.72	gel
100	191.6	42.7	6.81	51.44	3	0.32	18.57	t. g.
100	206	38.2	6.65	54.05	3	0.35	19.97	t. g.
100	221	33.6	6.49	56.62	3	0.37	21.42	t. g.
100	235	29.1	6.36	58.95	3	0.4	22.78	t. l.
100	250	24.5	6.22	61.31	3	0.42	24.24	t. l.
100	265	20	6.08	63.54	3	0.45	25.69	t. l.
100	280	15.4	5.95	65.69	3	0.47	27.14	t. l.
100	294	10.9	5.83	67.66	3	0.5	28.5	t. g.
100	79	90.4	7.91	24.66	2.8	0.13	7.66	gel
100	85.1	103.6	7.39	24.8	2.6	0.13	8.25	gel
100	92.2	118.9	6.86	24.94	2.4	0.13	8.94	-
100	295	14.04	5.77	67.12	2.95	0.49	28.6	t. g.
100	305	10.9	5.69	68.45	2.95	0.51	29.57	t. g.
100	285	9.79	5.98	67.19	3.07	0.49	27.63	t. g.
100	289	12.72	5.87	66.92	3	0.49	28.02	t. g.
100	300	9.09	5.78	68.45	3	0.51	29.08	t. g.
100	182	0	8.21	58.95	4.00	0.413	17.67	-
100	173	3	8.35	56.99	4.00	0.392	16.80	-
100	160	7	8.57	54.12	4.00	0.362	15.53	-
100	150	10	8.76	51.83	4.00	0.340	14.56	-
100	140	13	8.95	49.43	4.00	0.317	13.59	t. g.
100	121	19	9.33	44.51	4.00	0.274	11.75	gel
100	92	28	9.97	36.19	4.00	0.208	8.93	gel
100	76	33	10.36	31.08	4.00	0.172	7.38	gel
100	50	41	11.08	21.86	4.00	0.113	4.85	gel
100	30	47	11.72	13.87	4.00	0.068	2.91	-
100	94	0	11.48	42.58	5.00	0.266	9.13	gel
100	81	4	11.91	38.06	5.00	0.229	7.86	gel
100	68	8	12.37	33.19	5.00	0.193	6.60	gel
100	49	14	13.10	25.32	5.00	0.139	4.76	gel
100	36	18	13.66	19.40	5.00	0.102	3.50	gel
100	360	20	4.95	70.30	2.58	0.526	34.95	-
100	270	14	6.12	65.14	3.08	0.470	26.21	-
100	240	0	6.91	65.44	3.54	0.481	23.30	t. g.
100	200	12	7.41	58.49	3.55	0.402	19.42	-
100	170	21	7.84	52.57	3.55	0.342	16.50	-
100	140	31	8.28	45.75	3.54	0.280	13.59	t. g.
100	100	43	9.01	35.56	3.54	0.201	9.71	gel
100	70	52	9.65	26.65	3.55	0.141	6.80	gel
100	40	62	10.33	16.31	3.53	0.080	3.88	gel
100	200	131	5.14	40.59	2.00	0.226	19.42	-
100	169	141	5.34	35.62	2.00	0.191	16.41	-
100	140	150	5.55	30.64	2.00	0.158	13.59	-
100	100	162	5.87	23.14	2.00	0.113	9.71	-
100	60	100	8.09	19.14	2.75	0.093	5.83	gel
100	400	100	3.88	61.21	1.80	0.407	38.84	-
100	400	50	4.30	67.78	2.15	0.487	38.84	-
100	50	400	3.71	7.31	1.10	0.031	4.85	-
150	200	10	9.44	49.65	4.27	0.322	12.95	t. g.
150	180	16	9.74	46.11	4.27	0.290	11.65	t. g.
150	160	22	10.06	42.33	4.27	0.258	10.36	gel
150	140	28	10.40	38.29	4.28	0.226	9.06	gel
150	100	41	11.12	29.23	4.26	0.161	6.47	gel
150	83	46	11.47	25.04	4.27	0.134	5.37	gel
150	70	50	11.75	21.64	4.27	0.113	4.53	gel
150	60	53	11.98	18.91	4.27	0.097	3.88	gel
150	50	56	12.22	16.07	4.27	0.081	3.24	gel
150	38	60	12.50	12.49	4.27	0.061	2.46	-

* 25 w% stock solution was used. ** 15 % stock solution was used. h.v. refers to high viscosity solution; t.l. refers to turbid liquid (no gel, not viscous, but due to denaturation of proteins the solution is turbid); t.g. refers to turbid gel. - means no gel has been observed.

**Table A2:** EPR samples of Figure 4 and Figure A4.

Sample	20 wt% BSA (μL)	EtOH (μL)	H_2_O (μL)	26 mM SA (μL)	w% BSA	w% EtOH	x BSA 10^−5^	x EtOH	Ratio BSA:16 DSA
C	300	105	44.1	35	24.3	16.48	5	0.13	1:1
D	300	105	44.1	2*35	24.3	44.10	5	0.13	1:2
E	300	70	44.1	70	24.3	0	5	0.13	1:2
F	0	105	344	35	24.3	16.48	0	0.13	1:1

* Samples preparation of (A) and (B) are explained in their title.
